# A Multi-Dimensional Framework for Data Quality Assurance in Cancer Imaging Repositories

**DOI:** 10.3390/cancers17193213

**Published:** 2025-10-01

**Authors:** Olga Tsave, Alexandra Kosvyra, Dimitrios T. Filos, Dimitris Th. Fotopoulos, Ioanna Chouvarda

**Affiliations:** Laboratory of Computing, Medical Informatics and Biomedical Imaging Technologies, School of Medicine, Aristotle University of Thessaloniki, 541 24 Thessaloniki, Greece; aekosvyra@auth.gr (A.K.); dimfilos@auth.gr (D.T.F.); difoto@auth.gr (D.T.F.); ioannach@auth.gr (I.C.)

**Keywords:** data validation, cancer imaging, clinical metadata, harmonization, imaging data repository, multi-site derived data, data quality

## Abstract

**Simple Summary:**

In cancer imaging research, data collection, integration, and utilization to generate multicentric data repositories pose a series of significant challenges such as data harmonization, data quality, utility, and overall suitability for reuse. These challenges directly affect the reliability and robustness of research outcomes, making systematic approaches essential. This work presents the INCISIVE project approach for assessing the quality of cancer imaging and clinical (meta)data in a structured and transparent way. The proposed methodology serves as a guiding map to ensure the creation and maintenance of a high-quality data repository, which is a crucial factor for generalizable and trustworthy AI-services development and their safe adoption in healthcare practice.

**Abstract:**

**Background/Objectives:** Cancer remains a leading global cause of death, with breast, lung, colorectal, and prostate cancers being among the most prevalent. The integration of Artificial Intelligence (AI) into cancer imaging research offers opportunities for earlier diagnosis and personalized treatment. However, the effectiveness of AI models depends critically on the quality, standardization, and fairness of the input data. The EU-funded INCISIVE project aimed to create a federated, pan-European repository of imaging and clinical data for cancer cases, with a key objective to develop a robust framework for pre-validating data prior to its use in AI development. **Methods:** We propose a data validation framework to assess clinical (meta)data and imaging data across five dimensions: completeness, validity, consistency, integrity, and fairness. The framework includes procedures for deduplication, annotation verification, DICOM metadata analysis, and anonymization compliance. **Results:** The pre-validation process identified key data quality issues, such as missing clinical information, inconsistent formatting, and subgroup imbalances, while also demonstrating the added value of structured data entry and standardized protocols. **Conclusions:** This structured framework addresses common challenges in curating large-scale, multimodal medical data. By applying this approach, the INCISIVE project ensures data quality, interoperability, and equity, providing a transferable model for future health data repositories supporting AI research in oncology.

## 1. Introduction

Cancer remains one of the most significant health challenges globally, standing as a leading cause of death. In 2020, the World Health Organization (WHO) reported approximately 10 million deaths attributed to cancer [1]. Among the most prevalent types are breast, lung, colon, and prostate cancers, which continue to dominate the landscape in terms of newly diagnosed cases [2]. Despite these sobering statistics, advancements in early detection and personalized treatment approaches have shown immense potential to alleviate the global cancer burden [3]. In this regard, Artificial Intelligence (AI) and Machine Learning (ML) are emerging as transformative forces, with over two decades of application in (bio)medical research to address critical gaps in cancer detection, prognosis, and treatment [4]. The application of AI in cancer research has been catalyzed by several concurrent advancements, including sophisticated computational algorithms, high-performance computing systems, and the construction of extensive biomedical databases [5]. Together, these elements have laid the groundwork for the integration of AI and ML into numerous aspects of cancer care, ranging from early diagnosis and prognosis prediction to drug discovery and treatment response evaluation.

The availability of massive volumes of data has enabled the improved design, training, and optimization of AI models, which are increasingly serving as powerful tools to enhance patient outcomes, particularly in the areas of early diagnosis, personalized treatment, and clinical decision support. Data derived from diverse sources—such as Electronic Health Records (EHRs), Picture Archiving and Communication Systems (PACS), laboratory results, and genetic or multi-omics analyses—represent a rich foundation of clinical insight. However, the inherent heterogeneity, fragmentation, and multimodal nature of such data, often distributed across different healthcare systems, significantly hinder its effective utilization [6,7,8]. To fully harness the potential of AI, particularly in predictive modeling, this data must be carefully curated, harmonized, standardized, and made interoperable to ensure both accessibility and usability. Given these complexities, there is an urgent need for well-structured, high-quality, federated medical data repositories that can integrate datasets from multiple clinical centers under cohesive and governed frameworks. Moreover, variability in data quality, completeness, and formatting can compromise model performance and generalizability. Beyond accuracy, fairness and equity must also be prioritized. Biased training data will lead to biased results and unfair decisions, which is why carefully choosing diverse and representative data is essential for trustworthy AI in healthcare [9,10]. In this work, data fairness refers to the adequacy of data to be reliably combined and reused across different use cases. Fairness is handled as the balanced representation of key demographic and clinical subgroups, assessed for sex, age, cancer grade, and cancer type. This definition aligns with fairness principles in machine learning. Regarding trustworthy AI, the definition of the European Commission’s Ethics Guidelines for Trustworthy AI (2019) [11] is adopted, focusing on transparency (documented validation steps), accountability (traceable procedures), and robustness (data integrity and subgroup fairness). Challenges include data standardization due to inconsistent formats and terminologies across healthcare systems [12], data completeness issues from gaps in mandatory information [13], and privacy concerns where de-identification to protect patient confidentiality may also remove contextual details necessary for accurate AI predictions, such as diagnosis, age, ethnicity, or follow-up intervals. Data heterogeneity further complicates interoperability at both syntactic (data representation) and semantic (meaning interpretation) levels [14]. Additionally, sources of error in data collection involve biases from unbalanced demographic representation, leading to models that underperform on underrepresented groups, and selection bias if datasets are drawn from specific populations. Annotation errors can arise from human inaccuracies or inter-observer variability, while format inconsistencies occur due to differing imaging equipment and clinical systems [15]. Incomplete data may lead to biased models if not appropriately managed, and technical artifacts from equipment malfunctions or patient movement can compromise data quality and model performance.

Achieving this level of data quality necessitates rigorous (pre)validation processes and the development of advanced methodologies to detect any source of error and level of harmonization in diverse datasets. The 42-month EU-funded INCISIVE project (https://incisive-project.eu/) (accessed 15 August 2025) aimed to address many of these challenges with an aim to facilitate the widespread adoption of AI solutions in cancer imaging through a pan-European repository of cancer images. This repository, built on a federated approach, aggregates datasets from across European healthcare systems, enabling researchers and clinicians to leverage a critical mass of high-quality, harmonized data. A cornerstone of the INCISIVE initiative is the development of an AI toolbox designed to support various applications, including early diagnosis, patient stratification, and treatment response evaluation. By integrating these tools into clinical workflows, the project aimed to improve the efficiency and effectiveness of cancer care a step forward [16]. In this context, data quality and harmonization were considered a key pillar of the INCISIVE project given the multiplicity of data sources involved in terms of different cancer types, imaging modalities, and healthcare sites (participating hospitals and clinical centers acting as data providers). Standardized guidelines have been established to ensure that all data collected through the project adheres to a consistent structure and format. To achieve this, INCISIVE employed a rigorous pre-validation (prior to the pilot phase, AI services testing) framework to evaluate the quality and reliability of the datasets. The pre-validation phase served as an intermediary stage positioned between model development and clinical implementation/validation, acting as a critical checkpoint to bridge the gap between technical readiness and deployment [17]. In this context, pre-validation was not merely a preliminary review, but a structured, methodical process designed to detect inconsistencies, biases, or technical shortcomings within the data that could compromise downstream analysis or clinical applicability.

**Figure 1:**
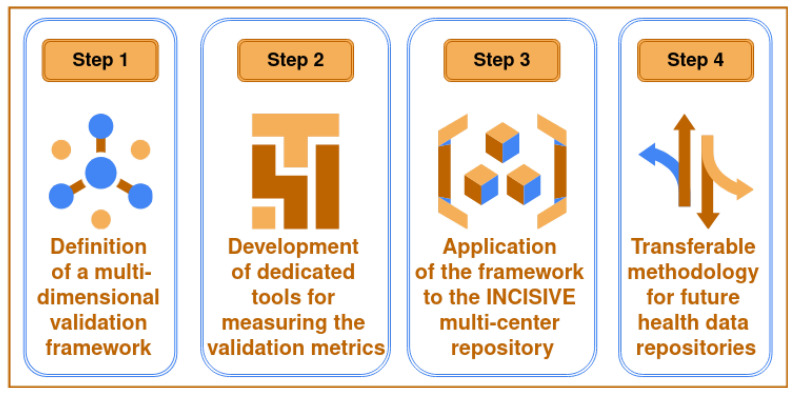
The Data Validation Procedure.

Recent studies have emphasized the importance of structured frameworks in the pre-validation phase of medical AI, proposing tools such as the METRIC framework [18] to systematically assess dataset quality with awareness dimensions that highlight key aspects relevant for AI in medicine. Such initiatives, along with harmonization checklists [19] and best-practice guidelines for imaging benchmarks [20], provide essential foundations for developing datasets that are fit for purpose, meaning they are sufficiently accurate, standardized, and well-documented to support integration across sites and to enable robust and trustworthy AI development. In the present work, the overall aim in pre-validation studies is to construct a comprehensive checklist or conceptual map to systematically identify errors and sources of variability, ensuring that datasets meet predefined quality benchmarks. This approach supports seamless data integration across sources and institutions by emphasizing harmonization, standardization, and high data reliability. As such, the pre-validation phase plays a pivotal role in laying the foundation for trustworthy, interoperable, and clinically meaningful data, ultimately facilitating a smoother and more robust transition into the pilot and subsequent clinical validation phases. Nevertheless, several important gaps exist. First, most existing frameworks treat clinical metadata and imaging data separately, and multimodal reviews continue to highlight the absence of unified, repository-level pre-validation pipelines capable of jointly inspecting metadata and pixel data across centers [21,22,23]. Second, fairness and subgroup balance are rarely evaluated systematically at the dataset stage, despite being critical for ensuring trustworthy AI; recent surveys and research articles call for explicit fairness evaluation and report persistent disparities in medical imaging AI [24,25], while new benchmarking efforts are only now emerging [26]. Third, implementation frameworks or tools remain limited for annotation-quality verification and for detecting DICOM images with burnt-in reports or protected health information, with only a few targeted OCR-based strategies recently proposed [27,28]. To address these gaps, this work contributes: (1) a multi-dimensional validation framework covering completeness, validity, consistency, integrity, uniqueness and fairness; (2) dedicated tools for measuring these metrics; (3) application of the framework to the INCISIVE multicenter repository; and (4) a transferable methodology for future health data repositories supporting AI in oncology. The procedure is depicted in Figure 1.

## 2. Materials and Methods

### 2.1. Background on INCISIVE Repository Data

**Figure 2:**
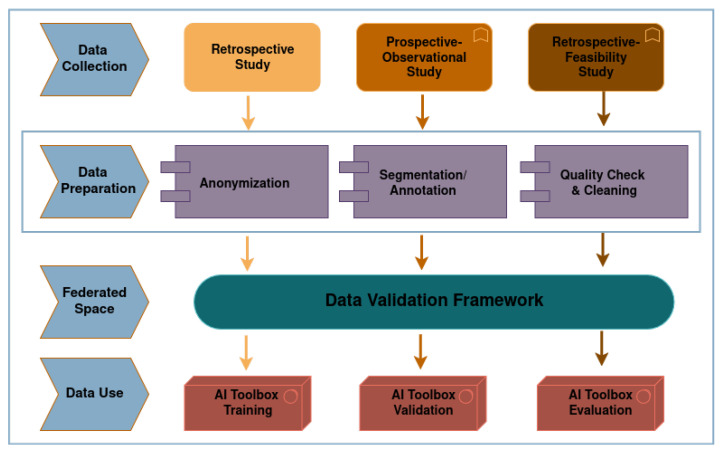
The Data Collection, Preparation, Validation and Usage Workflow in INCISIVE.

The INCISIVE project is focused on advancing cancer diagnostics and treatment monitoring for four cancer types: Lung, Breast, Colorectal, and Prostate. Briefly, the INCISIVE repository consists of two major components: the federated nodes (FNs) and the federated space. The FNs are decentralized data storage units within the Data Providers (DPs) premises, containing both data and procedure processors. This setup ensures that data remains local to each DP while still allowing collaborative analysis through federated learning. Meanwhile, the federated space serves as the central platform orchestrator, hosting all necessary operators for data processing and management, as well as the AI engines required for advanced diagnostics and research. This architecture enables seamless data integration and scalable processing across multiple sites while maintaining data privacy and security [29]. Based on clinical protocols, different imaging modalities and clinical data are employed at distinct time points in the patient journey, including diagnosis, treatment response assessment, and follow-up examinations. Imaging data were collected from hospital Picture Archiving and Communication Systems (PACS) [30] and stored as DICOM files. Depending on the cancer type, these images include CT, PET-CT, MRI, and Mammography (MG), X-rays [31]. To enhance the clinical value of the imaging data, expert radiologists manually segmented and annotated suspicious areas, including cancer lesion, in relevant images using ITK-SNAP [32], and these segmentations along with data were saved in NIfTI format. Additionally, clinical data was extracted from hospitals’ Electronic Health Records (EHRs) and organized into predefined templates (constructed based on medical experts’ guidance following cancer specific characteristics) to ensure uniformity and consistency across different sites. The data collection process was split into three distinct studies, namely, (a) A retrospective study which served the model training and development of the INCISIVE AI toolbox, (b) A prospective observational study for the validation of the INCISIVE AI-toolbox (comprising AI services that support more effective decision-making for healthcare professionals), and (c) A prospective feasibility study to serve the evaluation of the INCISIVE AI toolbox. Before uploading to the FNs, all collected data undergo a rigorous data preparation procedure, which includes de-identification, segmentation/annotation quality checks, and data curation/cleaning. This pre-processing ensures that the data not only complies with regulatory standards but also meets the requirements for effective integration and analysis within the federated environment. The INCISIVE data requirements are designed to maintain high-quality, standardized inputs suitable for AI model training and evaluation. These requirements cover various aspects, including the file types and folder structure, mandatory clinical data fields, image and annotation formats, and strict de-identification protocols to ensure patient privacy. To further support adherence to these standards and guarantee the quality and consistency of the integrated data, the Data Integration Quality Check Tool (DIQCT) was employed in the local side for data preparation before sharing data in the repository [33,34]. This tool automates the validation of data formats, checks for completeness, and ensures that all inputs are properly structured and de-identified before being made available for AI model training and evaluation. Figure 2 depicts the aforementioned workflow.

### 2.2. The Data Validation Framework

The validation strategy for the INCISIVE repository was designed to encompass both clinical and imaging datasets. For this, the validation process was structured into distinct methodological steps, each targeting specific aspects of data quality assessment. Data quality, in this context, refers to how well a dataset aligns with the requirements of its intended analytical use [35,36]. The evaluation focused on identifying typical data quality issues that could affect the dataset’s fitness for such use. These include:

- Data duplication
- Incompleteness
- Internal inconsistency
- Inaccuracies in recorded values
- Lack of standardized definitions
- Disorganized formatting or structure
- Deficiencies in data security

This structured approach provided a consistent basis for assessing and ensuring data integrity across diverse dataset types.

The proposed model is a multi-dimensional data validation framework designed to pre-validate multimodal clinical and imaging datasets. The model/approach integrates automated pipelines for deduplication (Uniqueness), annotation verification (consistency), DICOM metadata analysis (fairness), and anonymization checks (validity) for imaging data. It also computes standardized metrics referring to clinical data across five quality dimensions: completeness, validity, consistency, integrity, and fairness. These metrics are then aggregated into tabular outputs (Table 1), providing a transparent overview of dataset quality. By structuring the validation as a model, repeatability, scalability across institutions, and transferability to other repositories is ensured.

**Figure 3:**
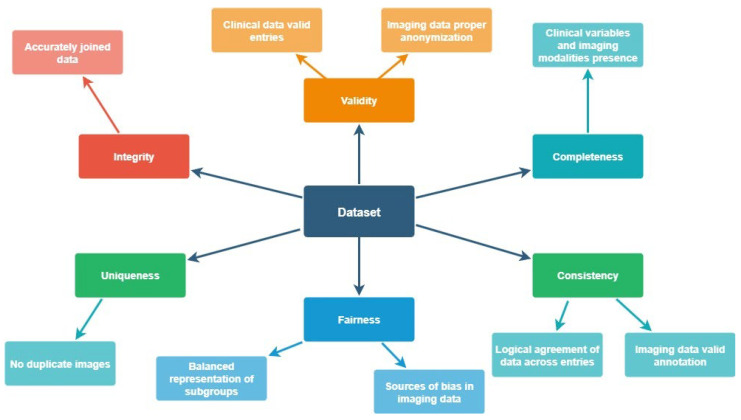
The Data Validation Framework for Clinical and Imaging Data.

Figure 3 depicts the data validation framework defined for both clinical and imaging data.

The proposed framework is in compliance with Article 78 of the European Health Data Space (EHDS) (corrigendum from European Parliament on 27 November 2024), on Data quality and utility label [37] which defines six dimensions that the data quality shall cover: completeness, uniqueness, accuracy, validity, timeliness and consistency. The framework also follows the suggestions of the QUANTUM initiative, a European-funded project under the HORIZON-HLTH-2023-TOOL-05-09 call on Tools and technologies for a healthy society, that started in January 2024 and aims to develop a Data Quality and Utility Label for the European Health Data Space [38]. Integrity is not listed in the dimensions that are proposed by the EHDS initiative; however, it was considered essential for our approach and added based on the DAMA’s definitions on data quality [39].

#### 2.2.1. Clinical Metadata Quality Assessment

In this study, the data quality assessment focused on clinical metadata and evaluated its ability to support project requirements across five dimensions, namely: 1. *Completeness*, 2. *Validity*, 3. *Consistency*, 4. *Integrity*, and 5. *Fairness*. Briefly, completeness measures the wholeness of the data, ensuring no gaps or missing information that would impair usability are detected. The evaluation methodology involved the identification of critical information in terms of essential data (marked as minimum mandatory essential information based on the predefined collection guidelines) elements that are required for the study. This mandatory information includes: (i) a set of mandatory fields, (ii) the existence of imaging modalities in one more timepoint beyond diagnosis. The dataset was then analyzed to detect any missing information, and the results were calculated and reported the percentage of complete records. It is worth mentioning that each patient was considered as a unique record. By the same token, validity assesses the extent to which data conform to predefined rules and value attributes established at the onset of the study. This dimension was evaluated based on data formats (for instance: specific structure, encoding, or organization of data that dictates how it is stored, processed, or exchanged), allowable types, and value ranges clearly defined. Again, the dataset was examined to identify valid information according to the established rules. The metric was reported as the percentage of records containing only valid values, with the record here being each inserted value. Similarly, consistency ensures uniformity of information across different sites, maintaining homogeneity. The approach for measuring consistency included the setting of uniform sets of information within the dataset. Such sets of information refer to consistency in cross-linked information within the dataset (such as reference of existence of laboratory examinations and actual insertion of laboratory results, reference of biopsy conducted and insertion of biomarkers identification). The dataset was analyzed to identify inconsistencies across different entries, and the metric was calculated and reported as the percentage of the matching values across the dataset. Integrity evaluates the accuracy of data references and linkages within the dataset. In this study, the focus was on integrating images with clinical metadata using a predefined template based on consensus and data collection guidelines, achieved by defining integration rules to successfully link images to metadata. (established using the aforementioned proposed template). These rules include proper naming of imaging modalities and proper link to the inserted to the template information with the provided modalities. The data were analyzed to verify proper integration, and integrity was quantified as the percentage of correctly integrated records, with records being the provided imaging modalities. Finally, fairness refers to the adequacy of data for reliable combination and utilization in diverse scenarios. Fairness was assessed by examining the distribution of data across key demographic and clinical subgroups, including sex, age group, cancer type, cancer grade per recruiting site. For each subgroup, we quantified representation balance and checked for systematic under- or over-representation that could bias subsequent AI model training. The methodology for evaluating this dimension aims to ensure the data’s adaptability and applicability across various use cases/scenarios

All rules that the data had to comply with to meet data quality requirements, as well as the rules used to calculate each metric, were provided to the data collectors in the form of standardized guidelines. These guidelines included detailed descriptions of every step in the data preparation process, along with the rules for data entry into the template, de-identification, and annotation. The methodology for defining this rule set and the resulting rule set are described in detail in previous work [34].

#### 2.2.2. Image Data Quality Assessment

##### Image Deduplication and Similarity Identification

A prevalent issue in data repositories is the presence of duplicate entries. Data/image deduplication is a critical process designed to remove redundant copies of data. The motivation to investigate whether duplicated images may appear in the repository originated from the fact that data collection is a manual process and therefore error-prone. Typical examples of errors that might lead to duplicated data include: a clinician accidentally placing the same imaging data under more than one patient (inter-directory analysis), or copying the same data into a different imaging series for the same patient. Additional possible sources of duplication include re-uploading the same study across different time points due to mislabeling of acquisition dates, data overlaps when the same patient is submitted by multiple clinical centers, partial duplication where only subsets of series from the same study are re-uploaded. As part of this work, a tool was developed/adapted to perform deduplication on a given dataset. Briefly, this tool accepts the directory path containing medical images from a DP as input and conducts a patient-specific analysis to identify and eliminate duplicate files. The process is carried out in two stages: initially, it examines each patient’s individual folder, comparing only the images within that folder. Subsequently, a comparison is made across all patient folders within a data provider’s directory. So, the approach was followed an (a) Intra-directory Analysis, and (b) Inter-directory comparisons. The core functionality of the tool relies on the difPy Python (python version 3.8) [40] package, which specializes in identifying duplicate images within a folder. For DICOM-format images, an initial conversion to a compatible image format was required. Given the computational expense of this conversion, it is currently applied only to directories containing modalities with a relatively low volume of DICOM images, such as in the case of Mammographies (MMGs).

##### Evaluation of Image Annotations

The annotation (the process of labeling medical imaging data) verification was conducted by evaluating the labels assigned to the segmentation masks, which were previously established as a consensus during the data collection phase, tailored to each cancer type [41]. These labels are not only associated with the specific cancer type but also with the imaging modality employed. For instance, in MMGs images, the data provider classifies each lesion into one of the following categories: (1) Benign, (2) Suspicious or Indeterminate, (3) Malignant, (4) Calcification, (5) Surgical clip, or (6) Axial lymph node. In contrast, for breast cancer MR images, the labeling system includes the categories: (1) Benign, (2) Suspicious or Indeterminate, and (3) Malignant. The number of the annotation labels was also counted for each cancer type and imaging modality to investigate whether the repository includes annotations for diverse cases. The full list of annotation types for each cancer type by modality is summarized in the Supplementary section (Table S1). In addition, an analysis of the consistency of the ROI and the number of slices between the annotation files and the imaging data was performed in order to evaluate whether an alignment between those modalities is required before their use. Finally, the existence of annotation files across different timepoints for each patient was investigated in order to assess whether the evolution of the lesion can be performed and thus use the repository for predictive modeling and access treatment effect.

##### DICOM Attributes/Tags Analysis for Imaging Data Sampling Bias

Moreover, data quality was also evaluated with respect to the DICOM tags that accompany each image as a potential source of bias that can lead to uniformity of the datasets. In so doing, several DICOM attributes which were considered to contribute to different images were analyzed. The selection of the herein presented attributes was based on literature sources [42,43] and guidance by experts in the field (HCPs, radiologists). The attributes were further split into categorical and numerical groups and are presented with respect to each cancer type/modality (MRI, PET-CT etc.) (full attribute selection in the Supplementary section). In particular, the attributes may be specific to different imaging modality, such as convolution kerel in CT, radiotherapy characteristics for PET or pulse sequence properties in MRI, or they might be independent to it, such as pixel spacing, slice thickness and ROI. In addition, the different vendor machines used for patient scanning were analyzed in terms of number of images and patients scanned for each vendor machine. The vendor, the field strength that was used for data acquisition, were some of the main aspects that contribute to different images. The evaluation was followed by statistical analysis, and the results are presented as median value with 1st and 3rd quartile.

##### DICOM Reports Detection

In this analysis, the whole dataset was systematically examined to identify images that contain DICOM reports. These DICOM reports stand as specialized images embedded within the image that summarize key details of the imaging examinations, often including patient information, imaging modalities, and diagnostic results. While the headers of these DICOM images can be de-identified using standard (dedicated state-of-the-art) de-identification tools, an important issue arises with “burnt-in” information [27]. This refers to patient-specific data that is directly embedded into the image pixels, rendering it inaccessible to traditional de-identification methods, which typically focus on metadata rather than image content. To address this challenge, a dedicated tool was developed to detect such images within the dataset. The tool specifically identifies instances where sensitive information may be embedded in the pixel data, utilizing Deid python package [44]. For the images that have been identified to contain embedded sensitive information, the results are further processed to identify if they do not contain a depiction of an organ based on the color spectrum of the image and the identified images are characterized as reports or not. This procedure enables the detection of images (reports) that require further processing or de-identification to ensure compliance with privacy and data protection standards. The results of the tool were validated through manual inspection. A subset of images was randomly sampled from the larger repository, and each was carefully reviewed to confirm the accuracy and reliability of the tool’s outputs. This approach enhances the accuracy of the de-identification process by addressing visual content, ensuring that the dataset maintains.

##### DICOM Anonymization Evaluation

An analysis of the images that did not conform to the de-identification profile defined in INCISIVE was performed. It is worth mentioning here that use of the quality tool which aimed to guide DPs for potential issues before data upload was not mandatory and thus, many providers might skip this step and they might upload data before the evaluation of the data de-identification process. In this respect, the analysis aimed to identify images which did not align with the de-identification protocol and they were uploaded to the repository. Any issue identified was communicated with the respective DP in order to proceed to the appropriate actions, before the actual use of the data from AI developers.

## 3. Results and Discussion

### 3.1. Clinical Data Validation

The data were evaluated in three separate phases that mirror the three distinct studies that took place during INCISIVE, namely retrospective, prospective (training) and observational. All values reported in the figures and tables were generated directly from the outputs of the proposed data validation framework. For each dataset, completeness, validity, consistency, integrity, and fairness were computed using predefined rules and automated scripts. Completeness was measured as the percentage of non-missing fields, validity as compliance with predefined value ranges and formats, consistency as agreement across repeated fields, and integrity as absence of duplicate or conflicting entries. Fairness values were derived from subgroup balance ratios across sex, age, cancer type, and cancer grade. The aggregated results per dataset and per study phase are summarized by metric in the following sections.

#### 3.1.1. Completeness

**Figure 4:**
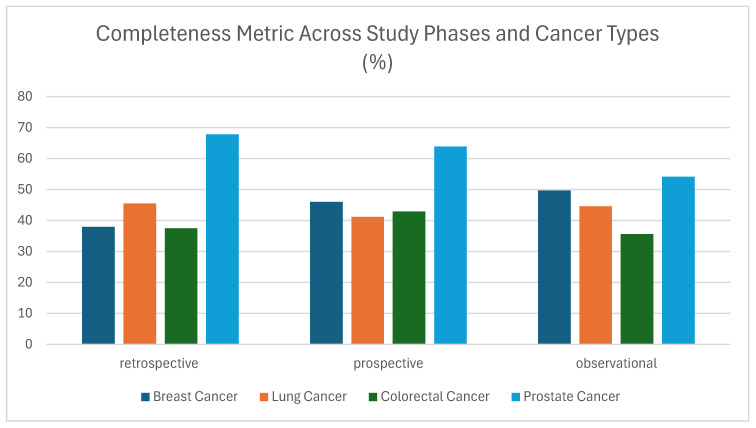
Completeness metric (%) across study phases and cancer types (breast, lung, colorectal, and prostate).

Regarding completeness, defined as the proportion of required data elements that were present and appropriately filled, revealed a more variable profile compared to other quality dimensions such as integrity (see below). In Figure 4, completeness values ranged from 35% to nearly 68%, with Prostate Cancer in the retrospective phase achieving the highest completeness at 67.79%, followed by Prospective Prostate Cancer at 63.87%. Other cancer types demonstrated more modest results, with Colorectal Cancer completeness falling below 43% in all phases, and particularly low in the observational phase (35.59%). Although values for Breast Cancer and Lung Cancer approached 50% in the observational phase, their overall performance remained moderate across the dataset. These findings indicate that completeness, unlike integrity, was more affected by study type and data source heterogeneity (Figure 4). In clinical and research data quality frameworks, completeness is a critical metric as it directly impacts on the usability of a dataset for analysis, modeling, or decision-making. Missing data can lead to bias, reduce statistical power, and limit generalizability, especially in multi-modal studies. Potential sources of error may be due to the fact that the majority of cases provided contain information only for the initial diagnosis: Patients participating in the study did not have a follow-up during the data collection; mandatory fields are not provided. Information not available in HER is also reported in [45]. The observed variation suggests that despite standardization efforts, data providers may have encountered challenges in populating optional or non-mandatory fields, or there may have been inconsistencies in interpreting which fields were required. Additionally, lower values in some cases may reflect the application of overly strict completeness rules, which did not always account for variations in clinical workflows, data availability, or study context. This further highlights the need for enhanced data curation strategies, more nuanced rule definitions, and possibly system-level changes such as data entry constraints, real-time completeness checks, or provider-specific feedback mechanisms. While completeness as a quality metric is often context-sensitive, depending on study design, data origin, and clinical pathways, its relatively low performance here points to a potential bottleneck in data quality that could affect downstream applications [46]. From a broader perspective though, these results reflect areas where data provider compliance may not have fully aligned with data collection expectations, especially in non-interventional or observational settings.

#### 3.1.2. Validity

**Figure 5:**
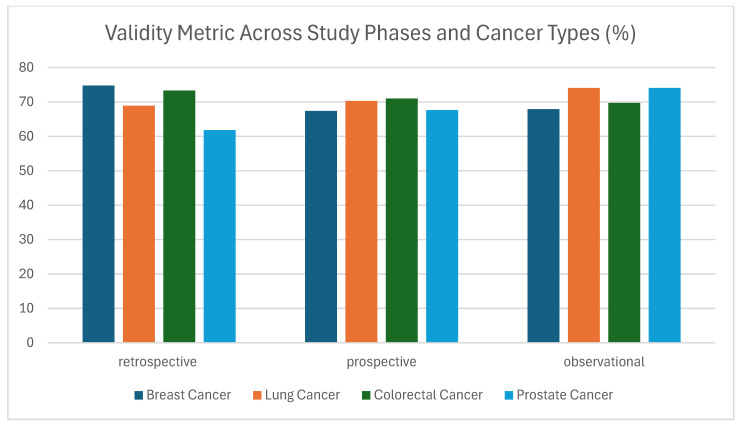
Validity metric (%) across study phases and cancer types (breast, lung, colorectal, and prostate).

By the same token, the validity metric, which evaluates the plausibility, clinical coherence, and conformity of data values to expected formats and standards, showed moderate-to-good performance across all study types and cancer domains. Validity scores ranged from 61.83% to 74.75%, with the highest observed in Breast Cancer (retrospective, 74.75%) and Prostate Cancer (observational, 74.07%), while the lowest was recorded in Prostate Cancer during the retrospective phase (61.83%) (Figure 5). These results suggest that while overall validity is acceptable, variations exist that may reflect inconsistent data entry, system heterogeneity, or weak enforcement of plausibility rules. Common issues impacting validity include inaccurate value ranges, or use of non-standard codes [47]. Particularly in the prospective and observational phases, the differences may be caused by how the data was recorded or by the lack of automatic checks during data entry. Higher values in Retrospective studies (breast & colorectal) derive from the correction done by the data collectors after the first measurement of the metric.

#### 3.1.3. Consistency

**Figure 6:**
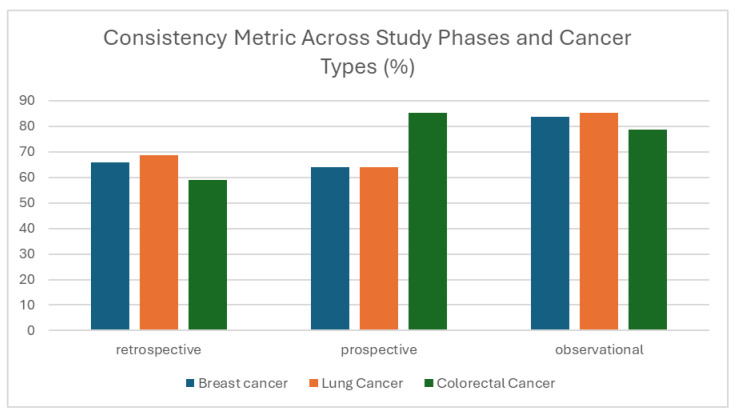
Consistency metric (%) across study phases and cancer types (breast, lung, colorectal, and prostate).

Similarly, consistency metric evaluates how uniformly data is structured and represented, especially across repeated entries or similar fields. The results showed clear variation across study phases. Prospective and observational datasets demonstrated high consistency, with scores ranging from 78.74% to 85.12% across all cancer types. In contrast, the retrospective phase showed notably lower values, particularly in Colorectal Cancer (59.09%) and Breast Cancer (65.83%) (Figure 6). This difference likely reflects improvements in data standardization and entry procedures introduced during the prospective (training) and observational phases. In retrospective data, inconsistencies often happen because the information was collected in the past, before common rules or formats were in place. By contrast, prospective and observational data likely benefited from predefined templates, clearer documentation, and more structured data entry approach. The low consistency score likely results from the fact that this specific check was not included in the user-side quality control tool (see above), allowing inconsistencies to go undetected during data entry.

#### 3.1.4. Integrity

**Figure 7:**
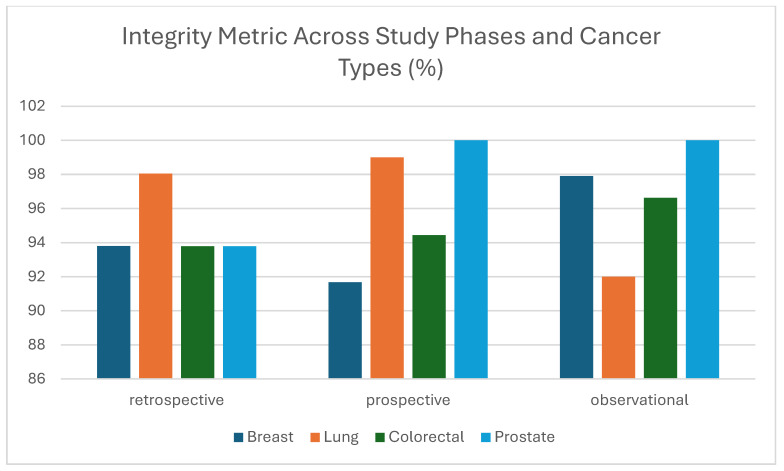
Integrity metric (%) across study phases and cancer types (breast, lung, colorectal, and prostate).

The evaluation of the integrity metric which is defined as the accuracy of data references and linkages within each dataset demonstrated consistently high performance across all study types (retrospective, prospective, and observational) and cancer types (Breast, Lung, Colorectal, and Prostate) (Figure 7). As shown in Figure 7, integrity values exceeded 91% across the board, with several cases, such as Prostate Cancer in the prospective and observational phases achieving 100%. Similarly, Lung Cancer data in the prospective phase and Breast Cancer data in the observational phase reached over 97%, indicating strong conformance with reference structures and linkage protocols. Although integrity as a standalone data quality metric can be context-dependent, it is a well-recognized and essential component within broader data quality frameworks, particularly in healthcare and clinical research. These consistently high scores not only reflect the reliability of the datasets but also highlight the success of training, structured templates, and adherence to collection protocols by data providers. Low integrity can be due to Mismatch in image count, such as providing 3 images when 4 were declared; Modality inconsistencies, like declaring MG (mammography) in the template but submitting CT images; Incorrect naming, where imaging files do not follow the required naming conventions. The high integrity observed suggests strong compliance with data structuring guidelines, underscoring the effectiveness of standardized processes and documentation. However, low integrity isn’t just a technical issue but a systemic red flag indicating underlying weaknesses in the data pipeline from collection to curation, which necessitates targeted interventions such as SOP revisions, staff training, and enhanced data governance.

#### 3.1.5. Fairness

Data fairness was evaluated across four dimensions: age, sex, cancer type, and cancer grade, with results reported separately for breast, colorectal, lung, and prostate cancers (Table 2, Table 3, Table 4, Table 5, Table 6, Table 7, Table 8, Table 9, Table 10, Table 11, Table 12, Table 13, Table 14 and Table 15). Fairness, in this context, refers to the adequacy of data representation across subgroups to ensure that datasets can be reliably combined, reused, and generalized across clinical applications and AI services development.

In the case of Breast cancer, age distribution was broadly representative, covering patients from 30 to 90 years (Table 2). However, some younger age groups (under 30) were underrepresented. Cancer grade data showed a marked imbalance, with grade 2 dominating the distribution for some data providers while grades 1 and 3 were comparatively sparse (Table 3). Cancer type was also unevenly distributed, with invasive ductal carcinoma (IDC) accounting for most cases across institutions (Table 4). Male breast cancer cases were recorded also, consistent with epidemiological expectations and indicating that rare subgroups were not entirely excluded (Table 5).

In the case of Colorectal cancer, age groups ranged similarly from 30 to 90 years, aligning with known incidence patterns (Table 6). Cancer grade (Table 7) data was missing for a few partners (data provider), while others provided a more balanced distribution across grades 1 to 3. The cancer type variable was heavily imbalanced toward adenocarcinoma (Table 9). Sex distribution (Table 8) appeared balanced across centers, with comparable numbers of male and female cases, suggesting no major sex-related bias in the dataset.

For the Lung cancer case, datasets covered patients from 35 to 90 years of age (Table 10). As with other cancers, younger age groups were scarcely represented. Sex distribution (Table 13) showed a clear male predominance, particularly in observational datasets (e.g., 19 males vs. 1 female), highlighting a potential fairness concern in model development. Cancer type data showed significant imbalance, with adenocarcinoma as the most common type, while small-cell, squamous cell, and other types were rarely recorded (Table 12). Cancer grade was largely missing across all centers, except for a few entries (Table 11).

Lastly, for prostate cancer, as expected, sex data for prostate cancer included only male cases. Age representation spanned the 30–90-year range, with most cases falling within the expected age bands for this disease (Table 14). Cancer grade was well documented at several DPs, using the standard 1–5 grading scale (Table 15). However, in other cases DPs provided no grade data, perhaps suggesting local inconsistencies in documentation practices.

Overall, the fairness metric results revealed substantial variability in subgroup representation across cancer types and institutions. Several datasets demonstrated limited diversity in cancer type and grade, with underrepresentation or missingness in rare subtypes and high or low grades. In some cases, data gaps were site-specific, likely reflecting differences in documentation protocols or system capabilities. Fairness was generally adequate in terms of age and sex distribution for breast and colorectal cancers, while lung cancer showed a notable male predominance. The absence or imbalance of subgroup data (e.g., rare histological types or missing grade fields) could reduce model robustness and introduce bias. Overrepresentation of certain data groups, resulting from multi-source data collection, should be carefully monitored to avoid establishing or reinforcing bias. These findings further underline the need for harmonized data collection strategies and minimum subgroup representation thresholds to ensure that AI models trained on these datasets are fair, generalizable, and clinically applicable [48].

### 3.2. Imaging Data Validation

#### 3.2.1. Annotations Results

For each patient, more than one imaging series may have been annotated, and details regarding the number of series annotated per time point are provided in (Table 16). Regarding the consistency of the annotation masks and the imaging data, in terms of ROI or the number of slices, it was observed that in breast cancer, 33.4% of the annotation files were consistent with the imaging series, while for the remaining annotation files, inconsistencies in ROI or the number of slices were observed. However, this observation does not affect the usefulness of the annotations, as the coordinates of the masks and images, as described in the file metadata, can be used to programmatically align the two modalities. The consistency rate for lung cancer was 95.8%, for colorectal cancer, 58.7% of the annotations were consistent, and for prostate cancer, the consistency rate was 99.5%. This inconsistency is attributed to the way the annotations were performed. In particular, the clinician used a primary imaging series to perform the annotation; however, the same annotation could also be applied to different series, which might have different ROIs or numbers of slices while an additional step is required in order to align the imaging files with the annotations, based on the files metadata.

Additionally, a number of empty annotation files were identified, suggesting the absence of any regions requiring annotation. This characteristic of the repository allows, on the one hand, the inclusion of subjects as a control group when the annotation corresponds to the baseline examination, and on the other hand, the evaluation of the treatment process when the annotation corresponds to a follow-up period. In more detail, for breast cancer, 32.8% of the annotation files were empty. For colorectal and prostate cancer, the respective percentages were 51.6% and 95%. Interestingly, for lung cancer, 78.5% of the annotations were empty, which was mostly related to X-ray images, where it had been agreed that healthy subjects would be included in the study. For the remaining imaging modalities for lung cancer, the percentage of empty annotations was only 2.3%.

The table below (Table 16) provides an overview of the number of patients with respect to the number of time points where annotation files are available.

**Table 16:** Number of patients with available annotations across multiple timepoints for the same imaging modality (1 timepoint = baseline; 2 timepoints = first follow-up; 3 timepoints = second follow-up; etc.).

	Imaging Modality	1 Timepoint	2 Timepoints	3 Timepoints	4 Timepoints	5 Timepoints
Breast	CT	43	19	8	2	0
	FUSCT	125	11	1	0	0
	FUSPT	127	10	1	0	0
	MG	94	22	3	42	0
	MR	47	4	0	0	0
	US	44	5	1	1	0
Lung	CT	78	39	27	13	1
	FUSCT	283	21	5	0	0
	FUSPT	242	16	4	0	0
	Xray	1899	10	4	1	0
Colorectal	CT	66	16	3	2	0
	FUSCT	98	12	4	0	0
	FUSPT	91	15	1	0	0
	MR	28	16	4	23	0
Prostate	MR	420	0	0	0	0

Regarding the annotation labels, it was identified that the DPs followed the annotation guidelines and used the predefined labels for each imaging modality. Concerning the number of annotation files, it was observed that some imaging examinations were performed when there was a strong likelihood of malignancy, such as PET-CT examinations. This is reflected in the percentage of annotation files that segment malignant lesions rather than benign ones. On the other hand, in some screening examinations, such as mammography (MG or US), more patients with benign lesions were included. The table below (Table 17) provides more details on the number of annotation files across different imaging modalities and cancer types. Details for the number of series and the respective annotation label per timepoint are provided in the Supplementary Material (Table S1).

#### 3.2.2. Imaging Data Sampling Bias Analysis

The imaging data included in the INCISIVE repository were provided by different clinical sites across multiple countries. In particular, for breast cancer, data from six different providers were included, while for lung, colorectal, and prostate cancer, the data originated from six, five, and three clinical sites, respectively. This characteristic of the repository ensures the inclusion of data acquired using different equipment with diverse scanning options and clinical acquisition protocols, thereby reducing bias in the final repository. The table below presents the results of the distribution analysis for the most common scanning parameters across different imaging modalities involving consecutive slices, confirming the diversity of the images used with regard to the slice thickness and the pixel spacing. Regarding the ROI, it was observed that for CT and PET examinations, the data did not differ, whereas for MRI, diverse ROIs were used. However, as shown on the table (Table 18), data were acquired using diverse vendor scanners, contributing to the diversity of the data included in the repository. Regarding MR images, variations in magnetic field strength were observed, with 38% of colorectal cancer images acquired using a 1.5 T scanner and the remaining 62% with a 3 T scanner. For prostate cancer, 76% of the images were acquired using a 3 T scanner, while in breast cancer cases, the vast majority (98%) were acquired using 3 T scanners.

More details regarding imaging-specific parameters that affect image characteristics, such as the convolutional kernel in CT images, pulse sequence characteristics in MR imaging, and radiopharmaceutical properties in PET imaging, were also taken into account.

#### 3.2.3. Duplication-Image Similarity Detection

A two-level approach was followed to ensure the inexistence of duplicated images in the repository. Initially, a specific dicom attribute that is considered to be unique for different images was analyzed. Following this, an image similarity analysis based on deep learning methodology was adopted. More specifically, the initial screening of the repository to identify images sharing the same Media Storage SOP Instance UID (DICOM tag: (0002,0003)) revealed several instances of duplicated files. A closer analysis confirmed that some series were indeed duplicated and could appear at multiple time points for the same patient or as additional series for different patients. However, the majority of the identified duplications were not actual duplicates. Instead, during the de-identification process, this DICOM tag was altered, resulting in duplicated values for images from different imaging modalities. Therefore, this duplication-check step serves only as an initial rapid screening process for the repository, before applying more resource-intensive similarity tests using deep learning techniques.

To analyze image similarity within and across directories, we systematically aggregated the data to quantify the total number of comparisons performed. This included assessing the frequency of similar images detected, as well as mapping their distribution across various directories and directory pairs. By doing so, we aimed to understand patterns of similarity both within individual datasets (intra-directory) and between different datasets (inter-directory). The results presented below specifically pertain to lung imaging datasets, encompassing both prospective and retrospective data for an indicative data provider (DP).

##### Intra-Directory Analysis

**Figure 8:**
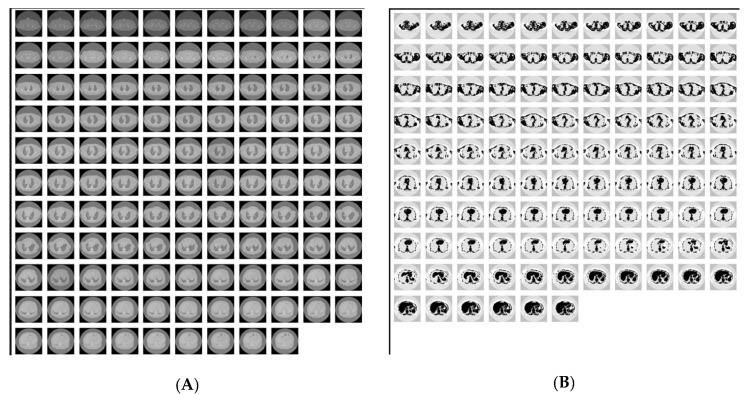
Representative example of a false positive in potential duplicate detection of CT examinations. (**A**) Shows the total number of comparisons performed, highlighting instances where similar images were detected, categorized by modality. (**B**) Displays the inter-directory comparison results specifically for the CT modality.

The intra-directory comparison was conducted with a “high” similarity grade, corresponding to an MSE threshold of 0.1. This stringent criterion was chosen to ensure that only images with very high degrees of similarity were flagged as similar/potential duplicates within the same folder (Table 19). To provide a summarized view of each case individually, we calculated the similarity percentage score, which represents the ratio of flagged potential duplicate occurrences to the total number of comparisons performed. Our analysis revealed a notably high number of similar images within PET/CT scans (FusCT/FusPT), often accompanied by high similarity percentages across most cases. This phenomenon can be attributed to the multi-dimensional imaging techniques used in PET-CT, which capture both metabolic and functional data of significant clinical value. However, due to their lower resolution compared to other medical imaging modalities and the reduced heterogeneity in the spatial context of organs as noted in prior studies, these scans require a more nuanced approach for similarity analysis, as also indicated by our results. For the relatively few cases involving CT modality, we conducted a preliminary investigation before validating our findings through a metadata-based analysis. Specifically, we examined cases where similar images were detected in inter-directory comparisons to assess whether false positives were also present in this modality. To ensure relevance, we focused on cases with high similarity percentages, selecting only the most likely instances of potential duplicates. Figure 8 illustrates two examples of the CT series. In our analysis, both were ultimately labeled as false positives, though their similarity percentages varied significantly. The first series showed a similarity score of 92.44% across 119 slices, while the second registered only 3.81% across 105 slices.

##### Inter-Directory Analysis

For the inter-directory comparison, a “high” similarity grade was used. Figure 8A illustrates the total number of comparisons performed and highlights the instances where similar images were detected, categorized by modality. This diagram reinforces the issue previously reported in the intra-directory analysis for PET-CT scans.

From the figure, it is evident that CT scans were the most frequently analyzed, with 578 comparisons. Fusion CT (FusCT) and Fusion PT (FusPT) followed with 54 and 22 comparisons, respectively, while X-rays had the fewest, with just 11 comparisons. As in our intra-directory analysis, we chose not to further investigate PET-CT scans. Instead, we focused on the CT modality, specifically examining its timepoint variations. Whereas Figure 8B, presents the inter-directory comparison results for the CT modality. The bar chart displays the number of comparisons performed and the detected potential duplicates, categorized by the time gap between CT examinations. The highest number of comparisons—and duplicate cases—occurred when scans were one timepoint apart. This likely results from follow-up exams reflecting similar clinical conditions, leading to false-positive duplicates, further amplified by standardized imaging protocols.

**Figure 9:**
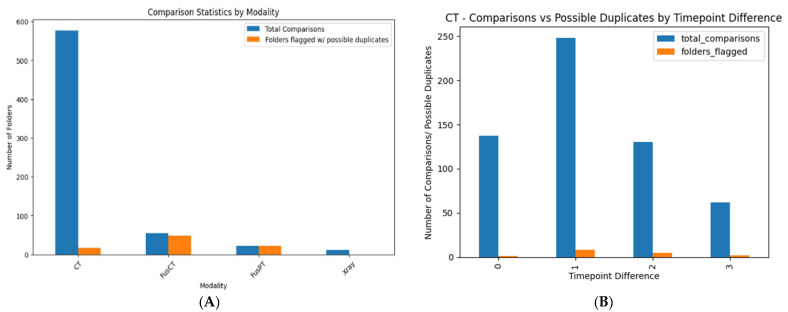
(**A**) Number of comparisons and possible duplicates by imaging modality. Most duplicates are found in CT. (**B**) CT-only: Comparisons and flagged duplicates by timepoint difference.

The experiment was then repeated using a “normal” similarity grade, with a higher MSE threshold. This more lenient criterion accounted for slight variations between images from different directories or patient cases. As expected, the number of duplicate cases increased for both CT and PET-CT scans, likely due to the system identifying more false positives under the relaxed similarity criteria (Figure 9).

Moreover, cases that involve possible duplicates identified from a comparison between two series which are one timepoint apart were also included. The similarity percentage recorded for this instance is 56%. In the images displayed, the tiles lined up on the left column are the same slice from the first series in the comparison, and the images on the right column are the slices that were flagged as possible duplicates from the second series. From the whole list of possible duplicates that were identified by the aforementioned approach, we randomly selected some cases to verify the existence of duplications in the repository. In one case it was found that the two series folders that were provided for the same patient, timepoint and imaging modality (CT) were flagged as possibly duplicated. From a closer view we observed that the images were not duplicates; however, they looked similar because they were acquired using different convolution kernels. The following example provides more information regarding the data that is included in the two series.

#### 3.2.4. Anonymization

The analysis of adherence to the de-identification protocol, as defined in the project, revealed that data providers (DPs) do not always follow the instructions. The identified issues can be grouped into two main categories. The first category relates to data that should have been hashed using the patient ID, such as the dates in the DICOM files. If the ID was not the expected one, the corresponding attribute was marked as problematic, since hashing was performed with an incorrect value. This issue is attributed to the fact that DPs either did not update the ID appropriately or placed the images in an incorrect folder. This finding does not affect patient de-identification, but it is related to data use and the association of the relative dates in order to identify which examinations related to the diagnosis and which one is related to the follow-up examinations. The correct dates can be found in the clinical data that are available for each patient and are provided in a tabular format. There may be various reasons for finding such anonymization misalignments in a large multicentric repository, for example, due to wrong use of the data preparation procedure. In any case, if such issues are detected, it is mandatory to be addressed as part of an early data curation phase, before making data available for wider use.

The second category concerns DICOM attributes whose values were expected to have been removed. In this case, it was found that 2000 images included gender information, and 2098 images retained age data. The removal of this information from the images was intended to avoid redundancy, and therefore, no identifiable information was provided. However, the fact that the de-identification protocol was not strictly followed highlights the need for quality checks as a mandatory step before making the data available, to ensure that no information that could lead to patient identification is shared.

#### 3.2.5. Identification of DICOM Reports

After running the analysis on the entire dataset, no instances of images with burnt-in DICOM reports were found. This indicates that no patient-specific information was embedded within the pixel data of the images. Given that no images containing embedded DICOM reports were detected, it can be concluded that the dataset complies with privacy and data protection standards as per the inclusion of such images. Therefore, no images required further erasure or de-identification and the validity metric for this case is 100%.

## 4. Conclusions

This work proposes a structured, multi-dimensional pre-validation framework as a practical and reproducible approach to ensure the readiness and trustworthiness of clinical and imaging data prior to their use in downstream applications, such as AI development and clinical research. Applied in the context of the EU-funded INCISIVE project, the framework was tested on multicenter datasets covering breast, colorectal, lung, and prostate cancers, evaluating both clinical metadata and imaging data quality. The results of this implementation demonstrate that the introduction of a dedicated pre-validation phase—distinct from model development or pilot testing—enabled the early identification and correction of structural, semantic, and referential issues. High integrity scores reflected the effectiveness of harmonized templates and standard operating procedures in ensuring reliable data linkages. Variations in completeness and validity highlighted the importance of robust record-keeping, the enforcement of mandatory fields, and automated quality checks. Improvements in consistency, particularly in the prospective and observational studies, underscored the impact of using standardized entry protocols. Fairness assessments revealed underrepresentation and missingness in key demographic and clinical subgroups, reinforcing the need for minimum inclusion thresholds and balanced data collection strategies across participating institutions.

On the other hand, the imaging validation component of the framework—focusing on deduplication, annotation quality, metadata consistency, and modality alignment—was critical to ensuring technical data soundness. The INCISIVE repository, built on a federated architecture and populated with harmonized, multi-institutional datasets, demonstrated a high degree of interoperability and data quality, making it a valuable resource for AI development in oncology. However, observed challenges—particularly in completeness and fairness—should be addressed to ensure that downstream models are both robust and generalizable. The results highlight that pre-validation should not be treated as a static checklist but rather as a foundational phase in the development of trustworthy, real-world AI solutions. Importantly, the insights gained through INCISIVE’s pre-validation process offer transferable guidance for future medical data repositories. They provide actionable evidence on how to assess and improve data quality and harmonization, particularly when datasets are destined for AI model development, evaluation, or fine-tuning. Several key findings and good practices emerged from this work. Validation studies conducted within INCISIVE exposed persistent issues around data trustworthiness, aligning with similar challenges reported in other large-scale imaging and clinical repositories [49]. The most substantial challenge was managing diverse data from multiple providers and cancer types, each contributing imaging modalities captured at various timepoints, under differing formats and standards.

Standardization during the design phase was essential. INCISIVE adopted well-defined templates tailored to each cancer type, with both mandatory and recommended fields selected based on literature review and expert clinical input. The use of structured guidelines such as TNM staging for cancer grading and a unified annotation vocabulary across providers ensured consistency in data collection and integration. Automated quality checks and tools, like the Data Integration Quality Check Tool (DIQCT), proved instrumental in identifying and resolving issues before data upload, particularly those related to structure, completeness, and de-identification. An iterative process of quality refinement, combining automated tools with manual review, enabled continuous improvement, ensuring that errors were corrected before they affected downstream use. Human factors such as manual data entry errors or deviation from format requirements were found to influence quality significantly. These were often caused by system limitations (e.g., missing values in EHRs) or misunderstanding of input constraints (e.g., using numeric values instead of coded fields). Training and education of data providers and clinicians proved essential. Within INCISIVE, dedicated annotation workshops helped clarify ambiguity, resolve inconsistencies, and improve coordination between radiologists and AI developers. Ongoing feedback mechanisms allowed dynamic communication between AI developers and data providers. Error reporting and resolution activities fostered a collaborative culture of quality monitoring. Finally, despite the application of rigorous validation strategies, real-world data challenges including incomplete fields, vendor-specific imaging variability, and imbalanced subgroup distributions were not fully avoidable. However, the INCISIVE showcased that these limitations can be mitigated through structured protocols, oversight, and continuous feedback. Collectively, the INCISIVE repository approach towards pre-validation showcases a practical and scalable model for constructing federated, high-quality medical datasets that are AI-ready. While certain areas require further optimization, particularly around subgroup representativeness and data completeness, the pre-validation framework implemented here enables reproducibility, enhances data reliability, and fosters equitable and transparent AI development.

## Figures and Tables

**Table 1:** Overview of the data quality assurance dimensions applied in the INCISIVE framework.

Dimension	Definition	Aim	Rule	Record	Metric	Example
Completeness	Wholeness of the data	Identification of gaps or missing information	Mandatory Information & Imaging modalities in diagnosis and one timepoint	Each patient	The percentage of records that are complete	No follow-up information, lack of information availability in EHR
Validity	Clinical data conformity to required value attributes	Identification of entries that do not follow predefined rules	Data format, allowable types & value ranges	Each value inserted in the dataset	The percentage of records in which all values are valid	Not confrontation with the defined rules (e.g., Stage: 3 instead of III)
	Anonymization	Identification of images that do not follow the de-identification protocol	DICOM metadata compared with the de-identification protocol	Each DICOM file	Percentage of DICOM files that do not follow the de-identification profile	DICOM metadata that should have been removed appear in the DICOM file
Consistency	Logical agreement of data across variables and records	Identification of inconsistencies within entries	Sets of entries (longitudinal, cross-linked)	Each value inserted in the dataset	The percentage of the matching values across the dataset	Erroneous cross-linked information (e.g., biological markers inserted, while the ‘Biopsy’ field is marked as ‘No Biopsy’)
	Annotation	Identification of annotation files that are consistent with the respective imaging data	Annotation ROI and number of slices coincide with the imaging data	Each annotation file	Percentage of annotation files that are consistent with imaging data	ROI in the annotation file and the number of slices are different from the respective imaging series data
Integrity	Accurately joined data references	Identification of inconsistencies in data references	Link between images and clinical metadata	Imaging modalities	The percentage of records properly integrated	Imaging examinations provided do not match insertions in clinical metadata template (e.g., MG was inserted while CT was provided, or 3 modalities were inserted while 4 were provided)
Uniqueness	No duplications or overlapping of values across all data sets	Identification of duplicate image files or series.	No duplicate images or series should be provided.	Images	The total number of identified duplicate image files or series within the dataset	Imaging examination series provided multiple times, Same patient imaging examinations provided as different timepoints.
Fairness	Balanced representation of subgroups relevant to trustworthy AI	Identification of classes balance	-	Target fields entries	The percentage of each class representation.	Comparable distribution across sex, age groups, cancer subtypes, etc.
	DICOM bias	Identification of imaging characteristics	-	Target imaging characteristics	Percentage of images that were acquired with specific imaging characteristic	Comparable slice thickness, pixel spacing, ROI or manufacturer scanner.

**Table 2:** Normalized Breast Cancer Age Distribution per DP.

Age Group	DP1 (%)	DP2 (%)	DP3 (%)
(20, 25]	0.0%	0.0%	0.0%
(25, 30]	0.0%	0.0%	0.0%
(30, 35]	3.57%	5.0%	0.0%
(35, 40]	7.14%	0.0%	0.0%
(40, 45]	0.0%	10.0%	8.7%
(45, 50]	14.29%	15.0%	4.35%
(50, 55]	7.14%	20.0%	0.0%
(55, 60]	10.71%	5.0%	8.7%
(60, 65]	14.29%	20.0%	21.74%
(65, 70]	10.71%	15.0%	21.74%
(70, 75]	10.71%	5.0%	17.39%
(75, 80]	17.86%	5.0%	0.0%
(80, 85]	3.57%	0.0%	13.04%
(85, 90]	0.0%	0.0%	4.35%
(90, 95]	0.0%	0.0%	0.0%

**Table 3:** Normalized Breast Cancer Grade Distribution per DP.

Data Provider	Grade 1 (%)	Grade 2 (%)	Grade 3 (%)
DP1	0.0%	25.0%	75.0%
DP2	0.0%	37.5%	62.5%
DP3	0.0%	77.27%	22.73%

**Table 4:** Normalized Breast Cancer Type Distribution.

Data Provider	IDC (%)	ILC (%)	IPLC (%)	IMC (%)	IUC (%)	IBC (%)	MPT (%)	SPC (%)	HBOCS (%)	UMN (%)	DCIS (%)
DP1	90.0%	10.0%	0.0%	0.0%	0.0%	0.0%	0.0%	0.0%	0.0%	0.0%	0.0%
DP2	1.96%	9.8%	0.0%	0.0%	0.0%	0.0%	0.0%	0.0%	0.0%	78.43%	9.8%
DP3	40.0%	20.0%	0.0%	20.0%	0.0%	0.0%	0.0%	0.0%	0.0%	0.0%	20.0%

**Table 5:** Normalized Breast Cancer Sex Distribution per DP.

Data Provider	Male (%)	Female (%)
DP1	0.0%	100.0%
DP2	0.0%	100.0%
DP3	0.0%	100.0%

**Table 6:** Normalized Colorectal Cancer Age Distribution per DP.

Age Group	DP1 (%)	DP2 (%)	DP3 (%)
(20, 25]	0.0%	0.0%	0.0%
(25, 30]	0.0%	0.0%	0.0%
(30, 35]	6.25%	7.14%	2.7%
(35, 40]	2.08%	0.0%	0.0%
(40, 45]	4.17%	7.14%	5.41%
(45, 50]	8.33%	0.0%	5.41%
(50, 55]	10.42%	0.0%	8.11%
(55, 60]	10.42%	0.0%	10.81%
(60, 65]	10.42%	7.14%	10.81%
(65, 70]	10.42%	14.29%	13.51%
(70, 75]	10.42%	21.43%	10.81%
(75, 80]	10.42%	28.57%	13.51%
(80, 85]	8.33%	14.29%	8.11%
(85, 90]	6.25%	0.0%	10.81%
(90, 95]	2.08%	0.0%	0.0%

**Table 7:** Normalized Colorectal Cancer Grade Distribution per DP.

Data Provider	Grade 1 (%)	Grade 2 (%)	Grade 3 (%)
DP1	57.14%	35.71%	7.14%
DP2	41.67%	50.0%	8.33%

**Table 8:** Normalized Colorectal Cancer Sex Distribution per DP.

Data Provider	Male (%)	Female (%)
DP1	57.5%	42.5%
DP2	50.0%	50.0%
DP3	58.75%	41.25%

**Table 9:** Normalized Colorectal Cancer Type Distribution per DP.

Data Provider	Adenocarcinoma (%)	Squamous (%)	Small-Cell (%)	Large-Cell (%)	Other (%)
DP1	88.24%	0.0%	0.0%	0.0%	11.76%
DP2	74.36%	25.64%	0.0%	0.0%	0.0%

**Table 10:** Normalized Lung Cancer Age Distribution per DP.

Age Group	DP1 (%)	DP2 (%)	DP3 (%)	DP4 (%)
(20, 25]	0.0%	0.0%	0.0%	0.0%
(25, 30]	0.0%	4.44%	0.0%	0.0%
(30, 35]	0.0%	2.22%	0.0%	0.0%
(35, 40]	0.0%	2.22%	5.26%	0.0%
(40, 45]	0.0%	4.44%	7.89%	0.0%
(45, 50]	0.0%	4.44%	7.89%	10.0%
(50, 55]	11.11%	6.67%	10.53%	0.0%
(55, 60]	22.22%	11.11%	10.53%	10.0%
(60, 65]	11.11%	11.11%	10.53%	0.0%
(65, 70]	0.0%	11.11%	13.16%	30.0%
(70, 75]	22.22%	11.11%	10.53%	30.0%
(75, 80]	33.33%	11.11%	10.53%	20.0%
(80, 85]	0.0%	11.11%	7.89%	0.0%
(85, 90]	0.0%	8.89%	5.26%	0.0%
(90, 95]	0.0%	0.0%	0.0%	0.0%

**Table 11:** Normalized Lung Cancer Grade Distribution per DP.

Data Provider	Grade 1 (%)	Grade 2 (%)	Grade 3 (%)
DP1	0.0%	100.0%	0.0%
DP2	0.0%	57.14%	42.86%

**Table 12:** Normalized Lung Cancer Type Distribution per DP.

Data Provider	Adenocarcinoma (%)	Squamous (%)	Small-Cell (%)	Large-Cell (%)	Other (%)
DP1	40.0%	40.0%	20.0%	0.0%	0.0%
DP2	12.5%	25.0%	0.0%	12.5%	50.0%
DP3	50.0%	30.0%	20.0%	0.0%	0.0%

**Table 13:** Normalized Lung Cancer Sex Distribution per DP.

Data Provider	Male (Normalized %)	Female (Normalized %)
DP1	100.0%	0.0%
DP2	60.0%	40.0%
DP3	28.57%	71.43%
DP4	62.67%	37.33%

**Table 14:** Normalized Prostate Cancer Age Distribution per DP.

Age Group	DP2_1 (Normalized %)	DP2_2 (Normalized %)
(20, 25]	0.0	0.0
(25, 30]	0.0	0.0
(30, 35]	4.17	0.0
(35, 40]	0.0	0.0
(40, 45]	4.17	0.0
(45, 50]	4.17	4.0
(50, 55]	8.33	16.0
(55, 60]	12.5	16.0
(60, 65]	20.83	20.0
(65, 70]	16.67	20.0
(70, 75]	12.5	16.0
(75, 80]	12.5	8.0
(80, 85]	4.17	0.0
(85, 90]	0.0	0.0
(90, 95]	0.0	0.0

**Table 15:** Normalized Prostate Cancer Grade Distribution per DP.

Cancer Grade	DP (Normalized %)
1.0	32.08
2.0	27.36
3.0	18.87
4.0	16.98
5.0	4.72

**Table 17:** Percentage of annotation labels that were marked as malignant (M) or benign (B). The total number of annotations areas are provided in each case (n).

	Breast	Lung	Colorectal	Prostate
CT	M: 29.6—B: 35.6 (*n* = 135)	M: 98.4—B: 1.6 (*n* = 310)	M: 73.8—B: 26.2 (*n* = 141)	
MR	M: 52.1—B: 22.8 (*n* = 215)		M: 68.2—B: 31.8 (*n* = 110)	M: 85.7—B:14.3 (*n* = 21)
FusCT	M: 77.1—B: 17.4 (*n* = 144)	M: 94.4—B: 5.6 (*n* = 339)	M: 97.5—B: 2.5 (*n* = 121)	
FusPT	M: 77.7—B: 17.8 (*n* = 146)	M: 94.4—B: 5.6 (*n* = 287)	M: 99.1—B: 0.9 (*n* = 110)	
XRAY		M: 24.4—B: 0 (*n* = 123)		
MG	M: 7.7—B: 22.9 (*n* = 1128)			
US	M: 46.6—B: 26.4 (*n* = 178)			

**Table 18:** Distribution of imaging characteristics across different modalities and cancer types. The results are provided as median [25% and 75% quartile].

		Slice Thickness	Pixel Spacing	ROI	Manufacturer
CT	Breast	3 [2 3.75]	0.98 [0.79 1.37]	512 [512 512] 512 [512 512]	GE: 46.6% Siemens: 46% Other: 7.4%
	Lung	2 [1.5 3.75]	0.98 [0.75 1.37]	512 [512 512] 512 [512 512]	GE: 45.3% Philips: 13.7% Siemens: 34.7% Other: 6.3%
	Colorectal	3 [1.5 5]	0.81 [0.73 0.98]	512 [512 512] 512 [512 512]	GE: 50.2% Siemens: 42.1% Philips: 6.2% Other: 1.5%
PT	Breast	3.27 [3.26 3.27]	2.74 [2.74 4.69]	256 [128 256] 256 [128 256]	GE:87.3% Siemens:12.7%
	Lung	3.26 [3.26 3.27]	2.74 [2.74 2.74]	256 [256 256] 256 [256 256]	GE: 81.6% Philips: 18.4%
	Colorectal	3.27 [3.27 5.00]	4 [2.74 4.1]	168 [144 256] 168 [144 256]	GE: 55% Siemens: 30% Philips: 15%
MR	Breast	7.5 [5.5 7.5]	0.68 [0.65 0.78]	512 [320 512] 512 [448 512]	GE: 1% Siemens: 98.5% Other: 0.5%
	Colorectal	6.6 [5 7.7]	1 [0.78 1.4]	288 [216 448] 320 [256 448]	GE: 1.8% Philips: 7.2% Siemens: 91%
	Prostate	3 [3 3.3]	1.63 [0.59 2.36]	136 [110 512] 160 [110 512]	GE: 20.8% Philips: 9% Siemens: 64.3% Other: 5.9%

**Table 19:** Results of the intra-directory comparison.

Total number of directories (patients) examined	60	
Total number of individual folders compared	436	
Instances similar images were found	186	
Comparisons count grouped by modality: (total number of examinations/number of folders similar images found)		
CT	237	35
FusCT	85	82
FusPT	69	69
X-ray	45	0

## Data Availability

Data used to support this research is part of the INCISIVE repository and can be accessed after application in the INCISIVE Data Sharing portal (https://share.incisive-project.eu/, accessed on 27 August 2024). It was approved by the Data Access Committee.

## Supplementary Materials

The following supporting information can be downloaded at: https://www.mdpi.com/article/10.3390/cancers17193213/s1, Table S1. The table summarizes the imaging modalities and annotation types included by tumor/cancer type.

## Abbreviations

The following abbreviations are used in this manuscript:

AI	Artificial Intelligence
CT	Computed Tomography
DICOM	Digital Imaging and Communications in Medicine
DIQCT	Data Integration Quality Check Tool
DP	Data Provider
HER	Electronic Health Record
FNs	Federated Nodes
HCP	Healthcare Professional
IDC	Invasive Ductal Carcinoma
INCISIVE	A European Project on AI in Cancer Imaging
ITK-SNAP	Interactive Toolkit for Segmentation of 3D Medical Images
MG	Mammography
ML	Machine Learning
MRI	Magnetic Resonance Imaging
NIfTI	Neuroimaging Informatics Technology Initiative
PACS	Picture Archiving and Communication System
PET	Positron Emission Tomography
ROI	Region of Interest
SOP	Standard Operating Procedure
UID	Unique Identifier
WHO	World Health Organization
X-ray	X-radiation
