# Supplementary material for: A Multi-Dimensional Framework for Data Quality Assurance in Cancer Imaging Repositories
Source: Cancers (Basel). 2025 Oct 1;17(19):3213. doi: 10.3390/cancers17193213 (PMC12524141; doi:10.3390/cancers17193213)
Supplement: Supplementary file 1 [file cancers-17-03213-s001.zip › cancers-3772345-supplementary.pdf]

Supplementary Materials: A Multi-Dimensional Framework for Data Quality Assurance in Cancer Imaging Repositories

Olga Tsave, Alexandra Kosvyra, Dimitrios T. Filos, Dimitris Th. Fotopoulos and Ioanna Chouvarda

Table S1. The table summarizes the imaging modalities and annotation types included by tumor/cancer type.

Cancer type	*Imaging modality*	*Labels*
Breast	**MG**	**Benign**
		**Suspicious or Indeterminate**
		**Malignant**
		**Calcification**
		**Surgical clip**
		**Axial lymph node**
	**MR**	**Benign**
		**Suspicious or Indeterminate**
		**Malignant**
	**CT**	**Benign**
		**Suspicious or Indeterminate**
		**Malignant**
		**Macrocalcifications**
	**FusCT/PT**	**Benign**
		**Suspicious or Indeterminate**
		**Malignant**
Lung	**CT/FusCT/PT**	**Benign**
		**Malignant**
	**Xray**	**Suspicious**
		**Problematic**
Colorectal	**MR**	**Benign**
		**Malignant**
	**CT**	**Benign**
		**Malignant**
		**Lymph Node**
	**FusCT/PT**	**Benign**
		**Malignant**
Prostate	**MR**	**Benign**
		**Malignant**
